# End-binding protein 1 stimulates paclitaxel sensitivity in breast cancer by promoting its actions toward microtubule assembly and stability

**DOI:** 10.1007/s13238-014-0053-0

**Published:** 2014-04-19

**Authors:** Youguang Luo, Dengwen Li, Jie Ran, Bing Yan, Jie Chen, Xin Dong, Zhu Liu, Ruming Liu, Jun Zhou, Min Liu

**Affiliations:** 1 State Key Laboratory of Medicinal Chemical Biology, College of Life Sciences, Nankai University, Tianjin, 300071 China; 2 Tianjin Key Laboratory of Medical Epigenetics and Department of Biochemistry, School of Basic Medical Sciences, Tianjin Medical University, Tianjin, 300070 China

**Keywords:** breast cancer, paclitaxel, microtubule, cell proliferation, apoptosis, end-binding protein 1, chemotherapy

## Abstract

Paclitaxel is a microtubule-targeting agent widely used for the treatment of many solid tumors. However, patients show variable sensitivity to this drug, and effective diagnostic tests predicting drug sensitivity remain to be investigated. Herein, we show that the expression of end-binding protein 1 (EB1), a regulator of microtubule dynamics involved in multiple cellular activities, in breast tumor tissues correlates with the pathological response of tumors to paclitaxel-based chemotherapy. *In vitro* cell proliferation assays reveal that EB1 stimulates paclitaxel sensitivity in breast cancer cell lines. Our data further demonstrate that EB1 increases the activity of paclitaxel to cause mitotic arrest and apoptosis in cancer cells. In addition, microtubule binding affinity analysis and polymerization/depolymerization assays show that EB1 enhances paclitaxel binding to microtubules and stimulates the ability of paclitaxel to promote microtubule assembly and stabilization. These findings thus reveal EB1 as a critical regulator of paclitaxel sensitivity and have important implications in breast cancer chemotherapy.

## INTRODUCTION

Microtubules are a component of the cytoskeleton and play a crucial role in diverse cellular activities. Microtubules are highly dynamic, and the dynamic properties are spatiotemporally regulated by microtubule-interacting proteins (Amos and Schlieper, 2005). End-binding protein 1 (EB1) is a key member of microtubule plus-end tracking proteins and is regarded as the master regulator at microtubule plus ends. EB1 tracks the plus ends of growing microtubules and thereby regulates microtubule dynamic instability (Akhmanova and Steinmetz, 2008; Schuyler and Pellman, 2001). EB1 promotes cell migration by stabilizing microtubules at the cell cortex and is also involved in mitotic progression (Asakawa and Toda, 2006; Li et al., 2011; Strickland et al., 2005; Wen et al., 2004). Recent studies point out that EB1 regulates microtubule dynamics through promoting persistent microtubule growth (Bieling et al., 2007; Li et al., 2011; Strickland et al., 2005; Wen et al., 2004; Zovko et al., 2008). EB1 is also a cofactor for many other microtubule-interacting proteins, which interact with EB1 directly or require EB1 for their efficient plus-end accumulation (Akhmanova and Steinmetz, 2008; Bieling et al., 2007; Dragestein et al., 2008; Galjart, 2010; Honnappa et al., 2009; Jiang and Akhmanova, 2011; Kronja et al., 2009).

Paclitaxel is a microtubule-targeting agent widely utilized in the clinic for the treatment of patients with breast cancer and several other solid tumors. Paclitaxel stabilizes microtubules and inhibits them from disassembly. As a result, chromosomes fail to achieve the metaphase spindle configuration, which further interferes with chromosome separation and leads to apoptosis (Blagosklonny et al., 2006; Dumontet and Jordan, 2010; Ikui et al., 2005). Paclitaxel has offered substantial improvement in patient survival, but the drug is not effective to all clinical cases. No useful predictive marker exists because of the limited understanding of the mechanisms regulating paclitaxel sensitivity (Dumontet and Jordan, 2010). Recent studies suggest a function of microtubule-interacting proteins in modulating cancer cell sensitivity to microtubule-targeting drugs, although the precise molecular mechanisms remain elusive (Mohan et al., 2013; Rouzier et al., 2005; Sun et al., 2012; Veitia et al., 2000; Wagner et al., 2005; Wang et al., 2009). Our previous study has demonstrated an oncogenic function of EB1 in breast cancer and show that its expression varies in different cancer cell lines (Dong et al., 2010; Sun et al., 2008). These findings prompt us to explore the potential role of EB1 in regulating paclitaxel sensitivity in breast cancer and the underlying molecular mechanisms.

## RESULTS

### EB1 expression in breast tumor tissues correlates with tumor response to paclitaxel-containing chemotherapy

**Figure 1:**
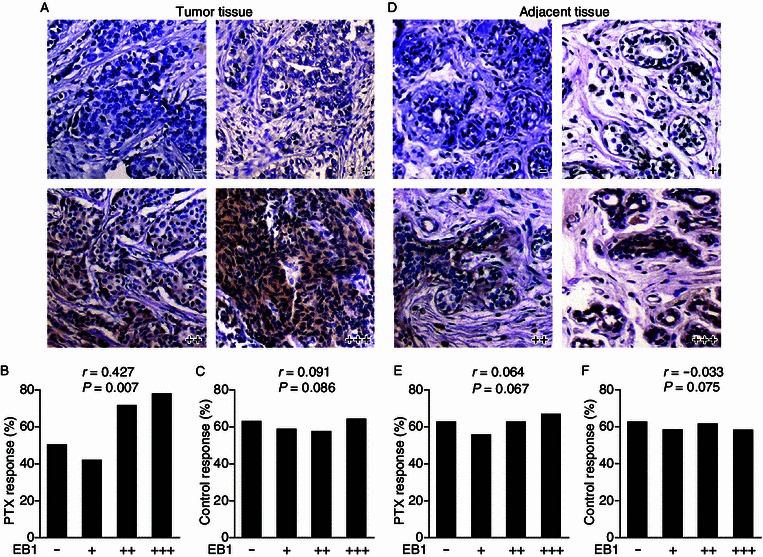
**EB1 expression in breast tumor tissues correlates with tumor response to paclitaxel**. (A) Immunohistochemical analysis of EB1 expression in breast carcinoma tissues. (B) Correlation analysis of EB1 expression in tumor tissues with tumor response to paclitaxel-containing chemotherapy. (C) Correlation analysis of EB1 expression in tumor tissues with tumor response to control chemotherapy. (D) Immunohistochemical analysis of EB1 expression in tissues adjacent to tumor. (E) Correlation analysis of EB1 expression in adjacent tissues with tumor response to paclitaxel-containing chemotherapy. (F) Correlation analysis of EB1 expression in adjacent tissues with tumor response to control chemotherapy. The percentage of response to the treatment was quantified as the number of responders divided by the number of total patients. The correlation between EB1 expression and the pathological response was examined by Wilcoxon rank sum test. *r*, correlation coefficient; *P*, statistical significance

To study the relationship between EB1 and paclitaxel sensitivity, we acquired tumor tissues from breast cancer patients who received neoadjuvant chemotherapy and then underwent surgical resection. Among these patients, 54 cases received paclitaxel-containing chemotherapy, and 45 cases received a control regimen without paclitaxel. We examined EB1 expression in tumor tissues and tissues adjacent to tumor by immunohistochemistry. The samples were classified into four groups according to EB1 expression, including negative staining (−), low staining (+), medium staining (++), and high staining (+++) (Fig. 1A and 1D). We found that EB1 expression varied significantly among patients. Out of the total samples, 18 were EB1-negative (10 received paclitaxel-containing chemotherapy and 8 received control treatment), 24 had low staining (12 received paclitaxel-containing chemotherapy and 12 received control treatment), 28 had medium staining (16 received paclitaxel-containing chemotherapy and 12 received control treatment), and 29 showed high staining (16 received paclitaxel-containing chemotherapy and 13 received control treatment).

By analyzing the correlation of EB1 expression with the pathological response of tumors to paclitaxel-containing treatment, we found a significant positive correlation between them (*r* = 0.427, *P* = 0.007) (Fig. 1B). In contrast, there was no obvious correlation between EB1 expression and tumor response to control treatment (*r* = 0.091, *P* = 0.086) (Fig. 1C). Furthermore, EB1 expression in tissues adjacent to tumor did not correlate with tumor response to paclitaxel-containing chemotherapy (*r* = 0.064, *P* = 0.067) or to the control regimen (*r* = −0.033, *P* = 0.075) (Fig. 1E and 1F). These results suggest that EB1 expression in breast tumor tissues might predict tumor sensitivity to paclitaxel-based treatment.

### EB1 enhances paclitaxel sensitivity in breast cancer cells

**Figure 2:**
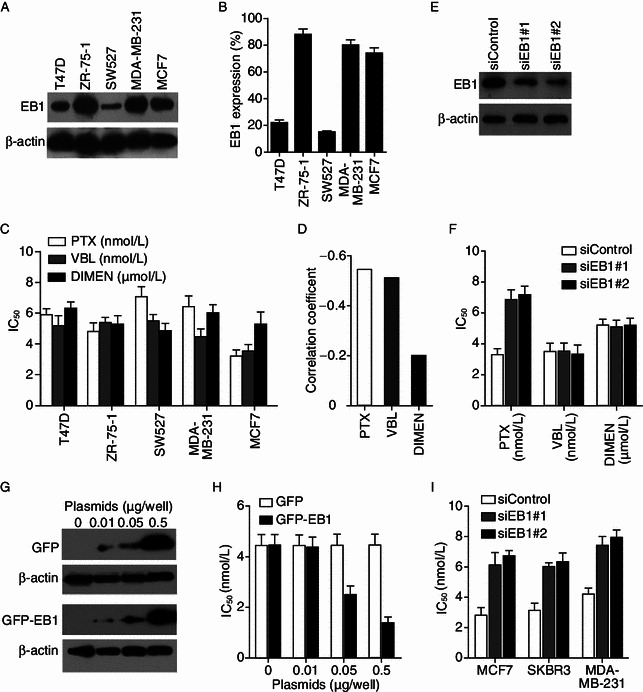
**EB1 expression increases paclitaxel sensitivity in breast cancer cell lines**. (A) Immunoblot analysis of EB1 and actin expression in breast cancer cell lines. (B) Experiments were performed as in (A), and relative EB1 expression was shown as the ratio of EB1 intensity to actin intensity quantified by densitometry. (C) Cancer cells were treated with paclitaxel (PTX), vinblastine (VBL), or dimethylenastron (DIMEN), and the IC_50_ values of the drugs were determined by sulforhodamine B staining. (D) Correlation analysis between EB1 expression in the cancer cell lines and the IC_50_ values of different drugs, examined by Spearman rank correlation test. (E) Immunoblot analysis of EB1 and actin expression in MCF7 cells transfected with control or two different EB1 siRNAs. (F) Cells transfected with different siRNAs were treated with paclitaxel (PTX), vinblastine (VBL), or dimethylenastron (DIMEN), and the IC_50_ values of the drugs were determined. (G) Immunoblot analysis of GFP, GFP-EB1, and actin expression in MCF7 cells transfected with different doses of GFP or GFP-EB1. (H) Cells transfected with different doses of GFP or GFP-EB1 plasmids were treated with paclitaxel, and the IC_50_ values of paclitaxel were then determined. (I) Cells transfected with control or EB1 siRNAs were treated with paclitaxel, and the IC_50_ values were determined by the MTT assay. Data shown in the graphs are means from three independent experiments

We then checked whether EB1 expression associates with paclitaxel sensitivity in breast cancer cell lines. We examined EB1 expression in T47D, ZR-75-1, SW527, MDA-MB-231, and MCF7 breast cancer cell lines by immunoblotting and then analyzed the expression level. Consistent with previous studies (Dong et al., 2010), EB1 expression levels varied among different cell lines; ZR-75-1, MDA-MB-231, and MCF7 cells displayed much higher EB1 levels than T47D and SW527 cells (Fig. 2A and 2B). Next, we examined the sensitivity of these cells to different drugs, including the microtubule-targeting agents paclitaxel and vinblastine, and a specific inhibitor of the microtubule-dependent mitotic kinesin Eg5, dimethylenastron (Liu et al., 2008), which was used as a non-microtubule-drug control. Cells were treated with the drugs at a series of concentrations and stained with sulforhodamine B, a dye that binds stoichiometrically to all cellular protein components, as a measure of cell proliferation (Vichai and Kirtikara, 2006). The IC_50_ value of each drug, which represents the drug concentration needed for inhibition of cell proliferation by 50%, was then calculated. T47D and SW527, which had low EB1 expression, showed relatively high IC_50_ values of paclitaxel and vinblastine (Fig. 2C). Intriguingly, MDA-MB-231 cells expressed abundant EB1, yet they were rather resistant to paclitaxel (Fig. 2A–C). Nevertheless, we found a remarkable negative correlation of EB1 expression with the IC_50_ values of paclitaxel (*r* = −0.546) and vinblastine (*r* = −0.512), but not with dimethylenastron (*r* = −0.198) (Fig. 2D). These results suggest that EB1 might increase the sensitivity of breast cancer cell lines to paclitaxel and vinblastine.

To gain more insights into the role of EB1 in regulating tumor cell sensitivity to microtubule-targeting drugs, we altered EB1 expression in MCF7 cells and then studied the change of drug sensitivity. MCF7 cells were chosen primarily due to their expression of a good amount of EB1 and their frequent use in the study of the mechanisms of action of microtubule-targeting agents. Cells were transfected with EB1 specific small interfering RNAs (siRNAs), which could knockdown EB1 expression efficiently (Fig. 2E), and stained with sulforhodamine B to analyze the IC_50_ values. EB1 siRNAs remarkably increased the IC_50_ values of paclitaxel, but did not obviously affect the IC_50_ values of vinblastine or dimethylenastron (Fig. 2F). These data indicate a specific effect of EB1 on paclitaxel sensitivity. We also transfected cells with different doses of GFP-EB1 or GFP and then analyzed the effect of EB1 overexpression on paclitaxel sensitivity. Overexpression of GFP did not affect the IC_50_ value of paclitaxel (Fig. 2G and 2H). In contrast, GFP-EB1 rendered cells more sensitive to paclitaxel as shown by decreased IC_50_ values (Fig. 2G and 2H).

We next investigated whether EB1 regulates paclitaxel sensitivity in cell lines that represent different types of breast cancer, including the estrogen receptor (ER)-positive cell line MCF7, the human epidermal growth factor receptor 2 (HER2)-positive cell line SKBR3, and the triple-negative cell line MDA-MB-231 (Neve et al., 2006). By MTT assays, we found that EB1 siRNAs increased the IC_50_ values of paclitaxel in all these cell lines (Fig. 2I). These data further demonstrate that EB1 enhances paclitaxel sensitivity in breast cancer cells.

### EB1 promotes the activity of paclitaxel to induce mitotic arrest and apoptosis

**Figure 3:**
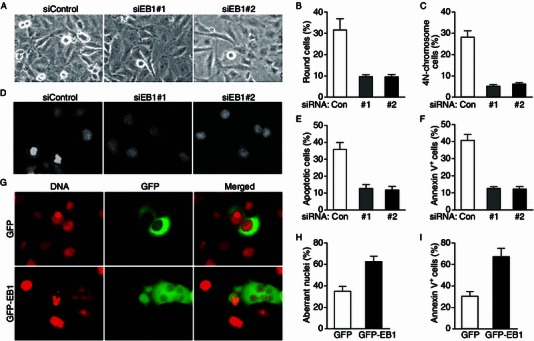
**EB1 promotes the activity of paclitaxel to induce mitotic arrest and apoptosis**. (A) MCF7 cells transfected with control or EB1 siRNAs were treated with 2 nmol/L paclitaxel for 24 h, and the images of cells were taken by phase contrast microscopy. (B) Experiments were performed as in (A), and the percentage of round cells was quantified. (C) MCF7 cells transfected with control or EB1 siRNAs were treated with 2 nmol/L paclitaxel for 24 h, and the percentage of cells having 4N chromosomes was quantified by flow cytometric analysis of DNA content. (D) MCF7 cells transfected with control or EB1 siRNAs were treated with 2 nmol/L paclitaxel for 48 h and stained with the DNA dye propidium iodide, and the images of cellular nuclei were taken. (E) Experiments were performed as in (D), and the percentage of apoptotic cells was quantified. (F) MCF7 cells transfected with control or EB1 siRNAs were treated with 2 nmol/L paclitaxel for 48 h and stained with PE-conjugated annexin V, and the percentage of annexin V-positive cells was quantified. (G) MCF7 cells transfected with GFP or GFP-EB1 were treated with 2 nmol/L paclitaxel for 48 h and stained with propidium iodide, and the images of cells were taken. (H) Experiments were performed as in (G), and the percentage of cells with aberrant nuclei was quantified. Aberrant nuclei refer to nuclei with abnormal morphology, such as fragmented nuclei or nuclei with irregular shapes. (I) MCF7 cells transfected with GFP or GFP-EB1 were treated with 2 nmol/L paclitaxel for 48 h and stained with PE-conjugated annexin V, and the percentage of annexin V-positive cells was quantified

We then sought to investigate the molecular mechanism of how EB1 regulates paclitaxel sensitivity. We analyzed mitotic arrest and apoptosis, two key cellular events following paclitaxel treatment (Jordan and Wilson, 2004). MCF7 cells transfected with control or EB1 siRNAs were treated with paclitaxel for 24 h, and the morphology of cells was photographed. The percentage of round cells was dramatically decreased by EB1 siRNAs as compared with control siRNA, indicating that EB1 siRNAs compromised the ability of paclitaxel to induce mitotic arrest (Fig. 3A and 3B). The decrease of paclitaxel-induced mitotic arrest by EB1 siRNAs was confirmed by the reduction of cells with 4N chromosomes following transfection of EB1 siRNAs (Fig. 3C).

By staining cells with the DNA dye propidium iodide, we further found that EB1 siRNAs remarkably inhibited the activity of paclitaxel to induce apoptosis (Fig. 3D and 3E). The inhibitory effect of EB1 siRNAs on paclitaxel-induced apoptosis was confirmed by quantification of the percentage of annexin V-positive cells (Fig. 3F). To verify the above effects of EB1, we analyzed the effect of EB1 overexpression on paclitaxel-induced apoptosis. Cells were transfected with GFP-EB1 or GFP and then treated with paclitaxel for 48 h. As shown in Fig. 3G and 3H, GFP-EB1 remarkably increased the ability of paclitaxel to induce the formation of aberrant nuclei, which is a characteristic of apoptosis. The enhancing effect of GFP-EB1 on paclitaxel-induced apoptosis was further confirmed by annexin V staining assay (Fig. 3I). Altogether, these results reveal that EB1 promotes the activity of paclitaxel to induce mitotic arrest and apoptosis.

### EB1 enhances the activity of paclitaxel to stimulate microtubule assembly and stabilization

**Figure 4:**
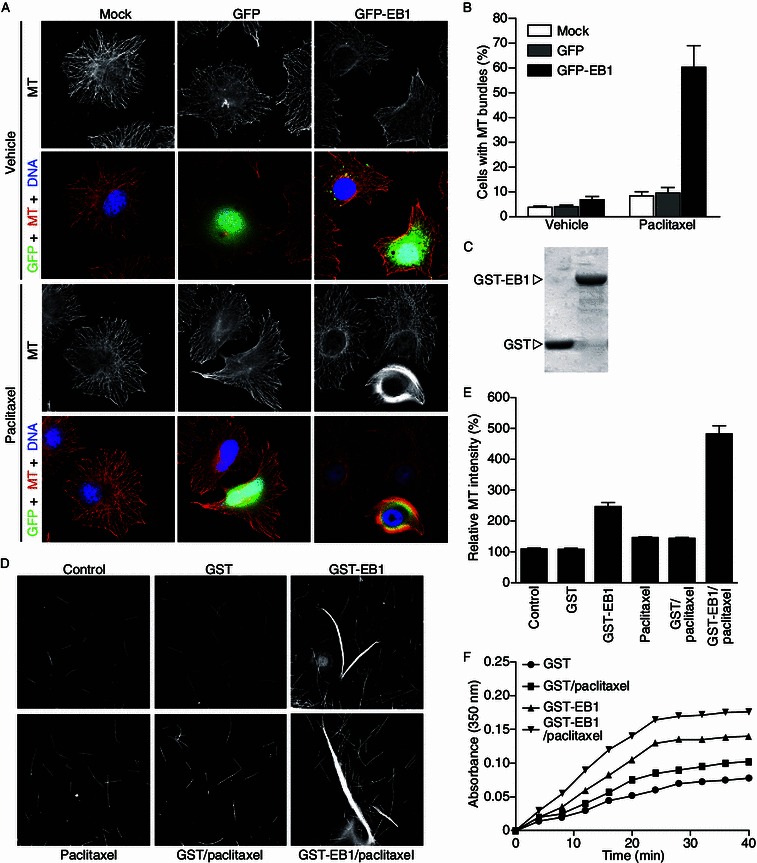
**EB1 enhances the activity of paclitaxel to stimulate microtubule assembly**. (A) MCF7 cells were transfected with GFP or GFP-EB1 and then treated with vehicle (DMSO) or paclitaxel (2 nmol/L) for 12 h. Cells were stained with anti-α-tubulin antibody and the DNA dye DAPI. (B) Experiments were performed as in (A), and the percentage of cells with microtubule bundles was quantified by counting 300 cells from 20 fields. (C) Coomassie blue staining of GST and GST-EB1 proteins purified from *E. coli*. (D) The mixture of MAP-free tubulin and rhodamine-labeled tubulin (9:1) was incubated at 37°C with purified GST or GST-EB1 in the absence or presence of paclitaxel. The morphology of polymerized microtubules was then analyzed under the fluorescence microscope. (E) Experiments were performed as (D), and the intensity of microtubules was measured and normalized to the control group. (F) MAP-free tubulin was incubated with purified GST or GST-EB1 at 37°C in the absence or presence of paclitaxel. The polymerization of tubulin into microtubules was examined by measuring the optical absorbance at 350 nm of wavelength

The activity of paclitaxel to trigger mitotic arrest and apoptosis results primarily from its effect on microtubule assembly and stabilization (Jordan and Wilson, 2004). To gain more mechanistic insight into the action of EB1 in modulating paclitaxel sensitivity, we transfected MCF7 cells with GFP or GFP-EB1 and then treated cells with paclitaxel (2 nmol/L). The percentage of cells with microtubule bundles was analyzed by immunofluorescence. As shown in Fig. 4A and 4B, GFP-EB1 significantly increased the ability of paclitaxel to induce microtubule bundles, as compared with GFP or the mock control.

To investigate the effect of EB1 on paclitaxel-mediated microtubule assembly *in vitro*, we purified GST and GST-EB1 from *E. coli* (Fig. 4C), and then analyzed their effects on microtubule assembly in the purified systems. A mixture of tubulin and rhodamine-labeled tubulin was incubated with purified GST or GST-EB1 in the presence or absence of paclitaxel at 37°C and the polymerized microtubules were analyzed under the fluorescence microscope. Consistent with previous studies (Bu and Su, 2001; Vitre et al., 2008), EB1 alone could modestly promote microtubule polymerization/bundling *in vitro*. Importantly, EB1 greatly enhanced the ability of paclitaxel to stimulate microtubule assembly/bundling (Fig. 4D and 4E). We also examined microtubule assembly *in vitro* over time by measuring the changes in optical absorbance at 350-nm wavelength. In agreement with the above findings, EB1 increased the ability of paclitaxel to induce microtubule assembly over time (Fig. 4F).

**Figure 5:**
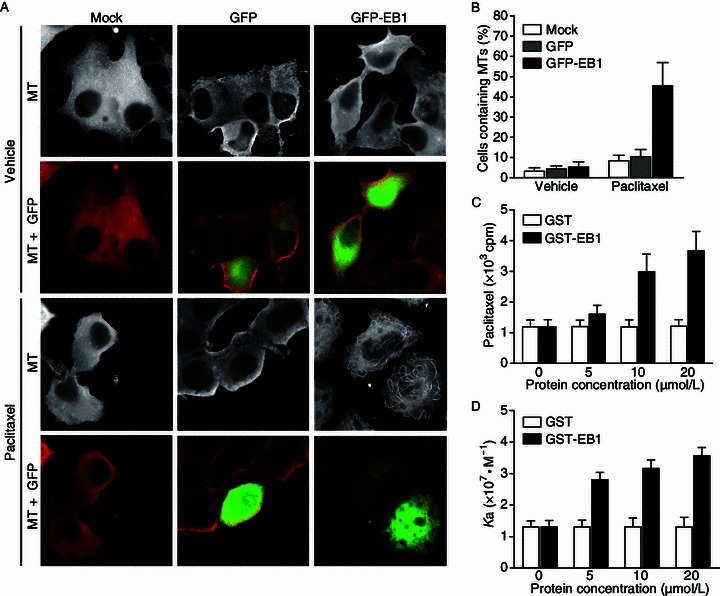
**EB1 increases the ability of paclitaxel to stabilize microtubules and stimulates paclitaxel binding to microtubules**. (A) MCF7 cells were transfected with GFP or GFP-EB1 and treated with vehicle (DMSO) or paclitaxel (2 nmol/L). Cells were then placed on ice for 30 min to depolymerize microtubules and stained with anti-α-tubulin antibody. (B) Experiments were performed as in (A), and the percentage of cells containing microtubules was quantified. (C) Purified GST or GST-EB1 at the indicated concentrations (μmol/L) on the x-axis was incubated with tubulin for 30 min at 37°C, and ^3^H-paclitaxel was added and incubated for another 30 min. Microtubules were pelleted by centrifugation and the radioactivity present in the pellet was measured. (D) Experiments were performed as in (C), except that gradient concentrations of ^3^H-paclitaxel were added to calculate the association constant (*K*a) between paclitaxel and microtubules as a measure of their binding affinity

Next, we sought to investigate the effect of EB1 on paclitaxel induced microtubule stabilization. MCF7 cells were transfected with GFP-EB1 or GFP followed by treatment with paclitaxel (2 nmol/L). Microtubules were then placed on ice for 30 min to depolymerize microtubules, and the percentage of cells containing microtubules was quantified to evaluate microtubule stability. We found that GFP-EB1, but not GFP, could greatly enhance the ability of paclitaxel to stabilize microtubules (Fig. 5A and 5B).

### EB1 promotes paclitaxel binding to microtubules

To understand the underlying mechanism of how EB1 increases paclitaxel-mediated microtubule assembly and stabilization, we investigated the influence of EB1 on the paclitaxel-microtubule interaction. We found that GST-EB1 could enhance paclitaxel binding to microtubules in a dose-dependent manner (Fig. 5C). To confirm the increase of the paclitaxel-microtubule association by EB1, we analyzed the association constant (*K*a) between paclitaxel and microtubules, as a measure of their binding affinity. As shown in Fig. 5D, GST-EB1 could increase the paclitaxel-microtubule association constant in a dose-dependent manner. These data thus reveal that EB1 promotes paclitaxel binding to microtubules.

## DISCUSSION

Microtubule dynamics are critical for many cellular activities, such as cell division, cell migration, and cell polarization. EB1 acts as a key microtubule plus-end tracking protein and regulator of microtubule dynamics (Akhmanova and Steinmetz, 2008). Many microtubule-interacting proteins, such as proteins with CAP-Gly domains or SxIP motifs, rely on EB1 directly or indirectly for their efficient localization at microtubules plus ends (Bieling et al., 2007; Dragestein et al., 2008; Honnappa et al., 2009; Kronja et al., 2009). Our previous studies have shown that EB1 plays an oncogenic role in the development of breast cancer (Dong et al., 2010). In the present study, we provide several lines of evidence demonstrating a novel function of EB1 in regulating cancer cell sensitivity to the microtubule-targeting agent paclitaxel: a) EB1 expression in breast tumor tissues correlates with the pathological response of tumors to paclitaxel treatment; b) EB1 expression correlates with paclitaxel sensitivity in breast cancer cell lines; and c) knockdown of EB1 expression decreases paclitaxel sensitivity in breast cancer cells and overexpression of EB1 has the opposite effect. Our findings suggest that the expression of EB1 might be used as a marker for the prediction of the pathological response to paclitaxel-based chemotherapy. In addition, our results suggest a possibility of improving the pathological response to paclitaxel through modulating EB1 expression.

We have also explored the potential mechanism of how EB1 regulates paclitaxel sensitivity in breast cancer cells. By alteration of EB1 expression, we find that EB1 could increase the activity of paclitaxel to induce mitotic arrest and apoptosis, two hallmark events in paclitaxel-treated cells. Although the casual relationship between paclitaxel-induced mitotic arrest and apoptosis remains controversial (Jordan and Wilson, 2004), our data suggest that EB1 increases the ability of paclitaxel to arrest cells at mitosis, resulting in increased formation of multinucleated cells and ultimately leading to enhanced apoptosis. By measuring the paclitaxel-microtubule association constant and performing microtubule-associated experiments in cells and *in vitro*, we find that EB1 increases the binding affinity between paclitaxel and microtubules and promotes paclitaxel-mediated tubulin polymerization and stabilization. Together, these findings indicate that EB1 promotes paclitaxel sensitivity in breast cancer cells by enhancing the ability of paclitaxel to stimulate microtubule assembly and stabilization and then cause mitotic arrest and apoptosis.

It is worthy of note that, besides EB1 expression, other factors such as tubulin subtypes and BCRA1 expression have also been implicated in paclitaxel sensitivity, with mechanisms involving paclitaxel-induced microtubule stabilization (Quinn et al., 2007; Tommasi et al., 2007). It is unclear currently whether EB1 exerts its effect independently or acts in concert with other factors in regulating paclitaxel sensitivity. In addition to the widely accepted notion that paclitaxel exerts its anti-tumor effect through promoting mitotic arrest in cancer cells and therefore cell death, there is also compelling evidence suggesting that another key mechanism of action of paclitaxel is promoting the activation of caspase signaling pathways, in which caspase 8 has been implicated (Komlodi-Pasztor et al., 2011). Thus, it would not be surprising if more mechanisms were discovered in the future as to how EB1 exerts its effect on paclitaxel sensitivity.

At present, it remains elusive how EB1 promotes paclitaxel binding to microtubules. It is possible that EB1 enhances the paclitaxel-microtubule interaction through structural or allosteric effects in a pattern similar to several other microtubule-binding proteins, such as Tau and CLIP-170 (Rouzier et al., 2005; Sun et al., 2012). In addition, the interaction between EB1 and paclitaxel in microtubule assembly may ensure microtubules in a proper structure, so more paclitaxel can bind to microtubules. Although our data show that EB1 promotes paclitaxel binding to microtubules *in vitro*, it is also possible that EB1 may increase the ability of paclitaxel to stimulate microtubule assembly via other mechanisms. For example, the stabilization of microtubule plus ends by EB1 may promote the action of paclitaxel toward tubulin polymerization into microtubules. Alternatively, given that EB1 acts as a loading factor for many other microtubule-interacting proteins in addition to its role as a regulator of microtubule dynamics (Akhmanova and Steinmetz, 2008; Honnappa et al., 2009), it is possible that EB1 may promote paclitaxel sensitivity in breast cancer cells through interaction with CLIP-170 or other microtubule plus end-tracking proteins. Considering the potential of EB1 as a marker to predict paclitaxel sensitivity or as a target to increase paclitaxel sensitivity, the molecular mechanism of how EB1 regulates paclitaxel sensitivity merits further investigation.

## MATERIALS AND METHODS

### Chemicals and antibodies

Paclitaxel, vinblastine, 4′,6-diamidino-2-phenylindole (DAPI), and sulforhodamine B were purchased from Sigma-Aldrich (St. Louis, MO, USA), phycoerythrin (PE)-conjugated annexin V was from Abcam (Cambridge, MA, USA), dimethylenastron was from Calbiochem (San Diego, CA, USA), and ^3^H-paclitaxel was from Moravek Biochemicals (Brea, CA, USA). Propidium iodide was purchased from Invitrogen (Carlsbad, CA, USA). Antibodies against EB1 (BD Biosciences, San Jose, CA, USA), β-actin and α-tubulin (Sigma-Aldrich), and GFP (Roche, Indianapolis, IN, USA), and horseradish peroxidase-conjugated secondary antibodies (Amersham Biosciences, Chandler, AZ, USA) were obtained from the indicated sources. Microtubule-associated protein (MAP)-free tubulin and rhodamine-labelled tubulin were from Cytoskeleton (Denver, CO, USA).

### Plasmids, proteins, and siRNAs

The mammalian expression plasmid for GFP-EB1 and the bacterial expression plasmid for GST-EB1 were constructed by cloning EB1 cDNA into the pEGFPN1 and pGEX6P3 vectors, respectively. The BL21 (DE3) strain of *E. coli* was used to express the proteins, and protein purification was carried out by using glutathione Sepharose 4B beads according to the manufacturer’s instructions (Promega, Fitchburg, WI, USA). EB1 and control luciferase siRNAs were synthesized by Ribobio (Guangzhou, China).

### Cell culture and transfection

T47D, ZR-75-1, SW527, MDA-MB-231, MCF7, and SKBR3 human breast cancer cell lines were cultured in RPMI 1640 medium supplemented with 10% fetal bovine serum at 37°C in a humidified atmosphere with 5% CO_2_. Plasmids were transfected into cells with the E-trans D reagent (Engreen, Beijing, China), and siRNAs were transfected with the Lipofectamine 2000 reagent (Invitrogen, Carlsbad, CA, USA).

### Tumor samples and pathological analysis

Breast carcinoma specimens were obtained from breast cancer patients who received neoadjuvant chemotherapy and then underwent surgical resection at Shanxian Dongda Hospital, Shandong, China. Of these patients, 54 were treated with a paclitaxel-containing regimen, and 45 were treated with a regimen without paclitaxel. Tumor tissues were obtained by surgical resection. To measure the pathological response of tumors, tumor specimens were cut into small pieces, fixed in formaldehyde, and embedded in paraffin. Sections were stained with haematoxylin and eosin and microscopically analyzed by an experienced pathologist for signs of tumor regression, mainly characterized by tumor necrosis, decreased tumor architectural detail, and replacement of tumor by fibrosis. The pathological response was defined by the proportion of histological changes in surgical specimens; responders showed histological changes in two-thirds or more of tumor tissues.

### Immunohistochemistry

For immunohistochemical analysis of EB1 expression, tissue sections were incubated with EB1 antibody and then with biotinylated secondary antibody and streptavidin-biotin-peroxidase. Diaminobenzidine was used as a chromogen substrate, and haematoxylin was used for counterstaining as described previously (Sun et al., 2013). EB1 expression level was graded based on the intensity of staining (0 = negative; 1 = low; 2 = medium; 3 = high) and the percentage of stained cells (0 = 0% stained; 1 = 1%–25% stained; 2 = 26%–50% stained; 3 = 51%–100% stained). A multiplied score (intensity score × percentage score) <2 was considered as negative staining (−), 2–3 as low staining (+), 4–6 as medium staining (++) and >6 as high staining (+++).

### Immunoblot analysis

Protein samples were separated by SDS-PAGE and transferred onto polyvinylidene difluoride membranes (Millipore, New Bedford, MA, USA). Then the membranes were blocked in 5% fat-free milk, and incubated sequentially with primary antibodies and horseradish peroxidase-conjugated secondary antibodies. Target proteins were visualized with enhanced chemiluminescence detection reagent according to the manufacturer’s instructions (Pierce Biotechnology, Rock-ford, IL, USA).

### *In vitro* cell proliferation assays

Cells were cultured in 96-well plates and treated for 48 h with different drugs with gradient concentrations. For the sulforhodamine B staining assay, cells were then fixed with 50% trichloroacetic acid, stained with 0.4% sulforhodamine B and washed with 1% acetic acid. The protein-bound dye was extracted with 10 mmol/L Tris base and the optical density at 490-nm wavelength was determined. MTT (3-(4,5-dimethylthiazol-2-yl)-2,5-diphenyltetrazolium bromide) assays were performed by using the Vybrant MTT Cell Proliferation Assay Kit (Thermo Fisher Scientific, Norcross, GA, USA) following the manufacturer’s instructions. The percentage of cell survival as a function of drug concentration was plotted to determine the drug concentration needed to prevent cell proliferation by 50% (IC_50_).

### Immunofluorescence microscopy

Cells grown on coverslips were fixed with 4% paraformaldehyde at room temperature or with methanol at −20°C, and washed with phosphate-buffered saline (PBS). The samples were blocked by incubation with 2% bovine serum albumin in phosphate-buffered saline. Cells were then stained with the DNA dye DAPI or propidium iodide or PE-conjugated annexin V. The coverslips were finally mounted with 90% glycerol in phosphate-buffered saline and examined with an Axio Observer A1 microscope.

### Flow cyometry analysis

Cells (2 × 10^6^) were centrifuged, washed twice with ice-cold PBS, and fixed in 70% ethanol. Cells were centrifuged at 1000 ×*g* for 10 min, and the supernatant was discarded. The pellets were resuspended in the phosphate/citrate buffer (0.2 mol/L Na_2_HPO_4_/0.1 mol/L citric acid, pH 7.5) at room temperature for 30 min. Cells were then washed with 5 mL of PBS and incubated with propidium iodide (20 μg/mL)/RNase A (20 μg/mL) in PBS for 30 min. Samples were analyzed on a Coulter Elite flow cytometer (Beckman Coulter, Inc., Fullerton, CA).

### *In vitro* tubulin polymerization assay

Spectrophotometer cuvettes held a mixture solution consisting of purified GST or GST-EB1 in the buffer containing 100 mmol/L PIPES, 1 mmol/L EGTA, 1 mmol/L MgSO_4_, and 1 mmol/L GTP. After the addition of MAP-free tubulin, the cuvettes were transferred to a temperature-controlled spectrophotometer and kept at 37°C. Tubulin polymerization was monitored by measuring the changes in absorbance at 350-nm wavelength as described previously (Huo et al., 2011; Sun et al., 2011). In another group of experiments, 10% rhodamine-labelled tubulin was added, and the polymerized microtubules were fixed, mounted onto slides, and examined with the fluorescence microscope.

### Microtubule sedimentation

Different doses of purified GST or GST-EB1 was incubated with 10 μmol/L MAP-free tubulin for 30 min at 37°C to induce microtubule assembly. ^3^H-paclitaxel was then added and the mixture was incubated for another 30 min. Microtubules were pelleted through a sucrose layer by centrifugation, and the radioactivity in the pellet was measured to analyze the interaction between paclitaxel and microtubules. To measure the association constant (*K*a) between paclitaxel and microtubules, the above experiments were performed with gradient concentrations of ^3^H-paclitaxel. The *K*a was then calculated by the following equation: *K*a = radioactivity in the microtubule pellet/(concentration of total tubulin used × radioactivity of total paclitaxel used).

## Abbreviations

DAPI: 4′,6-diamidino-2-phenylindole
EB1: end-binding protein 1
MAP: microtubule-associated protein
MTT: 3-(4,5-dimethylthiazol-2-yl)-2,5-diphenyltetrazolium bromide
PBS: phosphate-buffered saline
PE: phycoerythrin
siRNA: small interfering RNA
